# Melatonin-Nrf2 Signaling Activates Peroxisomal Activities in Porcine Cumulus Cell-Oocyte Complexes

**DOI:** 10.3390/antiox9111080

**Published:** 2020-11-03

**Authors:** Eui Hyun Kim, Muhammad Rosyid Ridlo, Byeong Chun Lee, Geon A. Kim

**Affiliations:** 1 Department of Theriogenology and Biotechnology, Research Institute for Veterinary Science, College of Veterinary Medicine, Seoul National University, Seoul 08826, Korea; hyun9214@snu.ac.kr (E.H.K.); rosyidridlodrh@gmail.com (M.R.R.); bclee@snu.ac.kr (B.C.L.); 2 Department of Bioresources Technology and Veterinary, Vocational College, Universitas Gadjah Mada, Yogyakarta 5281, Indonesia; 3 Department of Biomedical Laboratory Science, School of Medicine, Eulji University, 77, Gyeryong-ro, 771 beon-gil, Jung-gu, Daejeon 34824, Korea

**Keywords:** antioxidant, melatonin, Nrf2 signaling, oocyte, peroxisome, porcine, ROS

## Abstract

Melatonin and Nrf2 signaling synergistically improve mammalian oocyte maturation and embryonic development. Furthermore, previous studies have suggested an interplay between peroxisomes and Nrf2 signaling in cells, but it is still unclear whether peroxisomes are involved in oocyte maturation. The aim of the present study was to identify the possible roles of peroxisomes in the melatonin-Nrf2 signaling pathway during in vitro maturation (IVM) of porcine oocytes. Porcine oocytes were treated with melatonin (10^−9^ M) and brusatol, a Nrf2 specific inhibitor, in order to investigate the mechanism. Then, the rates of maturation and related gene and protein expression were analyzed. During oocyte maturation, melatonin upregulated the expression of gene and protein related to Nrf2 signaling and peroxisomal activities; RNA sequencing partially validated these results. Our results demonstrate that melatonin can activate Nrf2 signaling by binding to melatonin receptor 2, resulting in the upregulation of catalase. Moreover, peroxisomes were also found to be activated in response to melatonin treatment, causing the activation of catalase; together with Nrf2 signaling, peroxisomes synergistically prevented the generation of reactive oxygen species and enhanced oocyte quality. Thus, we suggest that a crosstalk might exist between Nrf2 signaling and peroxisomal activities in porcine oocytes.

## 1. Introduction

Melatonin (N-acetyl-5-methoxytryptamine) is a natural hormone that is produced by the pineal gland in mammals and it is primarily known to be involved in the regulation of circadian rhythm [1]. However, melatonin was found to play additional roles in processes, such as in steroidogenesis and mammalian reproduction [2,3]. Additionally, melatonin was also found to exhibit antioxidant effects against elevated levels of reactive oxygen species (ROS) [4,5] that are known to be responsible for the development of several pathological conditions [6]. Several studies in the field of animal reproduction indicate that melatonin regulates antioxidant effects in porcine oocytes and embryos by preventing ROS-induced DNA damage. Further, melatonin is also known to crosstalk with signaling pathways that are related to oocyte maturation, embryo development, and the activation of antioxidant-related factors [7,8,9]; additionally, melatonin significantly upregulates lipid metabolism by increasing fatty acid content and lipid droplet size in porcine oocytes [9,10]. Importantly, the antioxidant mechanism of melatonin is regulated by nuclear factor erythroid 2-related factor 2 (Nrf2) signaling [8,11,12].

Nrf2 mediates in several biological phenomenon, such as lipid metabolism [10], aging [13], and defense against oxidative stress in mammalian cells [14,15], including oocytes, and embryos [16,17]. Nrf2 mediates its antioxidant activity by translocating into the nucleus, where it binds to antioxidant response elements (AREs) to regulate the expression of genes related to the detoxication and antioxidant mechanisms [18,19]. Nrf2 signaling has been proposed to be essential for oocyte maturation and embryo development in cows [16], mice [17], and pigs [8], because it counteracts conditions that are generated in response to ROS exposure. Additionally, some of these studies verified the role of the Nrf2 signaling pathway by administering brusatol—a quassinoid from *Brucea javanica*—that is recognized as a specific inhibitor of Nrf2, thereby preventing the activation of genes whose expression is driven by the AREs, even in conditions of high oxidative stress [20,21]. Interestingly, peroxisome-related molecules have also been suggested to crosstalk with Nrf2 to protect against ROS-induced damage. Many publications indicate that Nrf2 and peroxisome-related molecules, such as peroxisome proliferator-activated receptor γ (PPARγ) function in a synergistic manner [22,23,24]. Some studies have proposed a strong relationship between PPARγ signaling and Nrf2 based on the fact that they exhibit synergistic effects against oxidative stress and lung injuries in mice [23,24]. However, no reports have investigated this crosstalk in mammalian oocytes, especially in pigs.

Peroxisomes are dynamic organelles that are present in most mammalian cells [25], and they are thought to play a significant role in mediating cellular defenses against ROS; this is attributed to the presence of catalase in these organelles [26,27], and the role of these organelles in ATP synthesis, lipid metabolism, and apoptosis [28]. Furthermore, Vázquez et al. suggested that melatonin induces peroxisome accumulation and enhances the activity of catalase [29], which is an H_2_O_2_-hydrolzing enzyme [30]. These findings indicate that Nrf2 signaling as well as melatonin influence the activities of peroxisomes. However, the effects of peroxisomal activities in oocytes remain largely unknown, although their effects in cells were found to be dynamic. Therefore, this study aimed to demonstrate the activation and biogenesis of peroxisomes and their related regulators—that are influenced by melatonin-Nrf2 signaling—in porcine cumulus-oocyte complexes (COCs).

## 2. Materials and Methods

### 2.1. Research Ethics and Chemicals

Porcine ovaries were delivered from a local slaughter house, after manufacturing processes. The Institutional Animal Care and Use Committee (IACUC) of Seoul National University approved the ovary usage (Approval ID: SNU-190621-2-1). All of the chemicals and reagents used in this study were obtained from Sigma–Aldrich Chemical Company unless indicated.

### 2.2. COC Retrieval and In Vitro Maturation (IVM)

The entire process of IVM was described previously [8]. Briefly, the ovaries were delivered from a local slaughter facility and then transferred to the laboratory at 38 °C. The retrieval of COCs was done by slicing the ovaries with sterilized surgical blades, then washed three times in washing medium. COCs with distinct cellular membrane, homogenous cytoplasm, and more than three layers of cumulus cells (CCs) were carefully selected, then they were cultured in IVM medium comprising M-199, 10 ng/mL, 10% porcine follicular fluid (*v/v*), 0.57 mM cysteine, 0.91 mM sodium pyruvate, epidermal growth factor, 10 μL/mL insulin-transferrin-selenium solution (ITS-A) 100× (Invitrogen, USA), 10 IU/mL human chorionic gonadotropin (hCG), and 10 IU/mL equine chorionic gonadotropin (eCG). The immature COCs were first cultured with hormones for 20–22 h, and then cultured again without the hormones for additional 20–22 h at 39 °C under 5% CO_2_ in 95% humidified air. Melatonin was dissolved in DMSO, then 10^−9^ M melatonin was used as the optimal concentration in accordance with previous studies [9,10]. For exact comparison, the control group was only treated with DMSO.

### 2.3. Cumulus Cell Expansion Assessment

The expansion rate of cumulus cell (CC) was evaluated after 42–44 h of IVM. In accordance with a previous study [31,32], CC expansion was assessed in the following manner: degree 0, the CCs are present as flattened monolayer with fibroblastic appearance and complete detachment from the oocyte; degree 1, CCs are spherical in shape and form a single layer; degree 2, expansion at only the outermost layer of the CCs; degree 3, expansion of all cell layers, except for the corona radiata, which are the most proximal to the oocyte; and, degree 4, the complete expansion of the CCs, including the corona radiata.

### 2.4. Parthenogenetic Activation (PA)

The entire process of PA was previously described [33]. After 44 h of IVM, COCs were denuded with 0.1% hyaluronidase. Denuded oocytes were washed and selected in Tyrode’s albumin lactate pyruvate (TALP) medium. Oocytes exhibiting the first polar body with distinct cellular membranes and homogenous cytoplasm were gradually equilibrated in activation medium containing 0.28 M mannitol, 0.1 mM MgSO_4_, 0.5 mM HEPES, and 0.1 mM CaCl_2_. The oocytes were transferred to the activation medium in a 3.2 mm double electrode chamber, then activated through 60 μs electric stimulation with a direct current pulse of 1.5 kV/cm using a BTX Electro-Cell Manipulator 2001 (BTX Inc., San Diego, CA, USA). Subsequently, the activated oocytes were washed and stabilized in porcine zygote Medium-5 (PZM-5; CSR-CK024; Waco Chemicals, Japan). Finally, they were transferred to 40 μL droplets of PZM-5, covered with mineral oil, and then cultured at 39 °C in a humidified atmosphere of 5% CO_2_, 5% O_2_, and 90% N_2_ for seven days.

### 2.5. Embryo Evaluation and Total Cell Count after PA

The day on which the activated oocytes were transferred to the in vitro culture (IVC) medium was denoted as Day 0. On Day 2 (48 h), embryos with even cleavage were observed under a stereomicroscope. Blastocyst formation was evaluated, and total cell numbers counted On Day 7 (168 h). The blastocysts from Day 7 were washed in PBS and then fixed for 1 h in 4% paraformaldehyde (*w/v*) in PBS at room temperature. Subsequently, the blastocysts were stained with 5 μg/mL of Hoechst 33342 for 8 min. After a washing process with PBS, the stained blastocysts were mounted on glass slides and then covered with cover slips. The total cell numbers of the blastocysts were counted under a fluorescence microscope (Nikon Corp., Japan) at 400× magnification.

### 2.6. Immunofluorescence Staining

The matured COCs were denuded with 0.1% hyaluronidase in TALP, and then washed in 1% PVA/PBS. The denuded oocytes were fixed with 4% paraformaldehyde (*w/v*) in PBS for 1 h at room temperature. Permeabilization of the oocytes were performed with 1% Triton X-100 (*v/v*) in distilled water (DW) for 1 h at 39 °C, washed four times in 1% PVA/PBS, and incubated in 2% BSA in 1% PVA/PBS for at 2 h to prevent non-specific bindings. Then, the oocytes were directly transferred to 2% BSA containing primary antibody for bone morphogenetic protein (BMP15) (1:200; PA5–34401; Thermo Fisher Scientific), as well as growth differentiation factor (GDF9) (1:200; ab93892; Abcam, USA), melatonin receptor 2 (MT2) (1:200; ARP64072_P050; Aviva Systems Biology, San Diego, USA), catalase [34] (1:200; 21260-1-AP; Proteintech, Rosemont, USA), NRF2 (1:200; 70R-50116; Fitzgerald Industries International, Acton, USA), phytanoyl-CoA hydroxylase (PHYH) (1:400; MBS3212923; MyBioSource, San Diego, USA), and peroxisomal biogenesis factor 19 (PEX19) (1:400; MBS9605735; MyBioSource, San Diego, USA), and performed the overnight incubation at 4 °C. After the incubation, the oocytes were washed several times in 1% PVA/PBS and then incubated with a secondary fluorescein isothiocyanate-conjugated anti-rabbit polyclonal antibody (1:200; ab6717; Abcam, Cambridge, UK) and Goat Anti-Rabbit IgG H&L (Texas Red^®^, 1:200; ab6719; Abcam, Cambridge, UK) at 37 °C for 2 h (light avoided). After the secondary antibody incubation and washing with 1% PVA/PBS, the counterstaining of the oocytes was immediately performed with 5 μg/mL Hoechst-33342 for 8 min. After through washing, they were mounted on glass slides with 100 % glycerol droplets, covered with cover slips, and then analyzed under a fluorescence microscope. The fluorescence was evaluated while using ImageJ software (version 1.46r; National Institute of Health, USA). Within three independent replications, at least 25 oocytes from each group were used for the staining.

### 2.7. ATP Content Assay

Matured COCs were denuded and washed in 1% PVA/PBS for three times, and then fixed in 4% PFA/PBS for 2 h at room temperature. Subsequently, the fixed oocytes were washed in 1% PVA/PBS droplets for three times and they were transferred to 0.5 μM of BODIPY FL ATP (BODIPY-ATP; A12410; Molecular Probes, Eugene, OR, USA) in PBS for 1 h at room temperature, light avoided. After the staining, the oocytes were again washed in PBS and mounted on glass slides, covered with slips. An epifluorescence microscope (TE2000-S; Nikon) was used for capturing images, and the intensities of ATP content was measured while using ImageJ software (version 1.46r; National Institutes of Health, USA). The intensities of the control group were standardized to 1. At least 20 oocytes from each experimental group were used for the staining.

### 2.8. Measurement of Intracellular Glutathione (GSH) and Reactive Oxygen Species (ROS) Levels

After 42–44 h of IVM, the matured COCs were denuded and washed several times thoroughly in TALP medium. H2DCFDA (2′,7′-dichlorodihydrofluorescein diacetates; Invitrogen) and CellTracker Blue (4-chloromethyl-6.8-difluoro-7-hydroxycoumarin; CMF2HC; Invitrogen) were used for measuring the intracellular ROS and GSH levels in oocytes, respectively. The oocytes were transferred to 10 μM of CellTracker Blue or 10 μM of H2DCFDA in TALP medium, and incubated for 30 min at room temperature, avoiding light. Stained oocytes were again washed several times in TALP medium, then they were transferred to a 4-μL droplet of TALP medium, covered with mineral oil. Epifluorescence microscope (TE2000-S; Nikon, Tokyo, Japan) was used for measuring the intensities of the fluorescence, observed through UV filters (460 nm for ROS and 370 nm for GSH), then the images were captured. Analysis was performed by Image J software and the intensities of the control group was standardized to 1. For three independent replications, at least 36 oocytes from each experimental group were used in this experiment.

### 2.9. mRNA Sequencing

The total RNA was used in order to construct cDNA libraries with the TruSeq Stranded mRNA LT Sample Prep Kit. The protocol consisted of poly A-selected RNA extraction, RNA fragmentation, random hexamer primed reverse transcription and 100nt paired-end sequencing by Illumina NovaSeq 6000. The libraries were quantified using qPCR according to the qPCR Quantification Protocol Guide and qualified using an Agilent Technologies 2100 Bioanalyzer. The raw reads were preprocessed from the sequencer to remove low quality and adapter sequence before analysis and aligned the processed reads to the *Sus scrofa (*Sscrofa11.1*)* using HISAT v2.1.0 [35]. HISAT utilizes two types of indexes for alignment (a global, whole-genome index and tens of thousands of small local indexes). These two types’ indexes are constructed while using the same BWT (Burrows–Wheeler transform)/ a graph FM index (GFM) as Bowtie2. The reference genome sequence of *Sus scrofa (*Sscrofa11.1*)* and annotation data were downloaded from the NCBI. Subsequently, the transcript assembly of known transcripts was processed by StringTie v1.3.4d [36,37]. Based on the result of that, the expression abundance of transcript and gene were calculated as read count or FPKM value (Fragments Per Kilobase of exon per Million fragments mapped) per sample. The expression profiles are used to undertake additional analysis, such as DEG (Differentially Expressed Genes). In groups with different conditions, differentially expressed genes or transcripts can be filtered through statistical hypothesis testing.

### 2.10. Analysis of Gene Expression by Quantitative Real-Time PCR

Matured COCs were denuded and then washed with PBS, then they were stored at −80 °C until RNA extraction. At least 400 COCs from each experimental group were used for RNA extraction by the RNAqueous^TM^ Micro Kit (Invitrogen, Vilnius, Lithuania). The quantified mRNA was measured using a NanoDrop 2000 Spectrophotometer (Thermo Fisher Scientific, Wilmington, DE, USA), and then complementary DNA (cDNA) was synthesized while using amfiRivert cDNA synthesis Platinum Master Mix 0 (genDEPOT, Houston, TX, USA), according to the manufacturer’s protocol. For quantitative real-time PCR (qRT-PCR), mixtures of each reaction contained 10 μL SYBR Premix Ex Taq (Takara, Otsu, Japan), 0.4 μL (10 pmol/μL) forward primer, 0.4 μL (10 pmol/mL) reverse primer, 8.2 μL of Nuclease Free Water (NFW), and 1 μL cDNA in a PCR plate (Micro-Amp Optical 96-Well Reaction Plate, Applied Biosystems, Singapore). Sequences of the primers are listed in Table 1.

The amplification was done by the Applied Biosystems StepOneTM Real-Time PCR Systems (Applied Biosystems, Waltham, MA, USA). With the following parameters, forty cycles of reactions were performed: denaturation for 15 s at 95 °C, annealing for 1 min at 60 °C and 1 min of extension at 72 °C. The oocytes were collected from at least three biological replicates and four technical replications were performed in a plate. Each target gene expression was quantified relative to that of the endogenous control gene (*GAPDH*). The relative expressions were calculated by the following equation [8]:

R = 2^−[^^ΔCt sample −^^ΔCt control]^ (1)

### 2.11. Western Blotting

More than 1000 COCs from each experimental group were sampled and stored at −80 °C until use, and then they were used for protein extraction with PRO-PREP^TM^ (Intron Biotechnology, gyeong-gi, Korea). The equal concentration of protein was determined by the Bradford reagent. The SDS sample buffer was used for lysing the samples (Protein concentrations were set as 20 μg/μL), and boiled for 10 min at 95 °C, then SDS-PAGE was conducted using 12% SDS-polyacrylamide gel electrophoresis in running buffer at constant 120V for 70 min. The proteins were then electrically transferred onto polyvinylidene difluoride membranes (Thermo Fisher Scientific) using a blotting apparatus adjusted to 350 mA for 60 min. Membranes were blocked with 5% (*w/v*) bovine serum albumin in Tris-buffered saline with 0.05% (*v/v*) Tween-20 (TBST) for 2 h at room temperature. The membranes were hybridized with first antibody against each of the proteins: Anti-β-actin (1:4000, ab6276; Abcam), NRF2 (1:500; 70R-50116; Fitzgerald Industries International), PEX19 (1:1000; MBS9605735; MyBioSource, USA), and PHYH (1:1000; MBS3212923; MyBioSource, USA) overnight at 4 °C in TBST. Thereafter, the membranes were washed with TBST and then incubated with second antibodies: Rabbit antimouse immunoglobulin G (IgG; (1:5000, ab6728; Abcam, UK) or Goat antirabbit IgG (1:5000, ab6721; Abcam, UK), for 2 h at room temperature. Subsequently, the membranes were washed three times by shaking in TBST for 30 min, and then detected using the enhanced chemiluminescence reagent (West-Q ECL solution, GenDEPOT), according to the manufacturer’s protocol. The images were developed with Fusion Solo software (Vilber Lourmat, France).

### 2.12. Statistical Analysis

At least three replications were performed in each experiment. GraphPad PRISM 5.01 (PRISM 5, GraphPad Software, Inc., San Diego, CA 92108, USA) was used for statistical analysis. In order to determine significant differences among experimental groups, data were expressed as the mean ± S.E.M, analyzed using unpaired t test and univariate analysis variance (ANOVA) with Tukey’s Multiple Comparison Test. *p* Values < 0.05 were considered to be significantly different among the experimental groups. In the case of RNA sequencing, the relative abundances of gene were measured in read count while using StringTie. We performed the statistical analysis to find differentially expressed genes using the estimates of abundances for each gene in samples. Genes with one more than zeroed read count values in the samples were excluded. The filtered data were log2-transformed and subjected to TMM Normalization. Statistical significance of the differential expression data was determined while using edgeR exactTest [38] and fold change in which the null hypothesis was that no difference exists among groups. False discovery rate (FDR) was controlled by adjusting the p value using the Benjamini–Hochberg algorithm. For DEG set, hierarchical clustering analysis was performed using complete linkage and Euclidean distance as a measure of similarity. Enrichment of gene ontology analysis was performed for DEGs using g:Profiler [39].

## 3. Results

### 3.1. Optimization of Brusatol Concentration and Subsequent Co-Treatment with Melatonin

**Figure 1:**
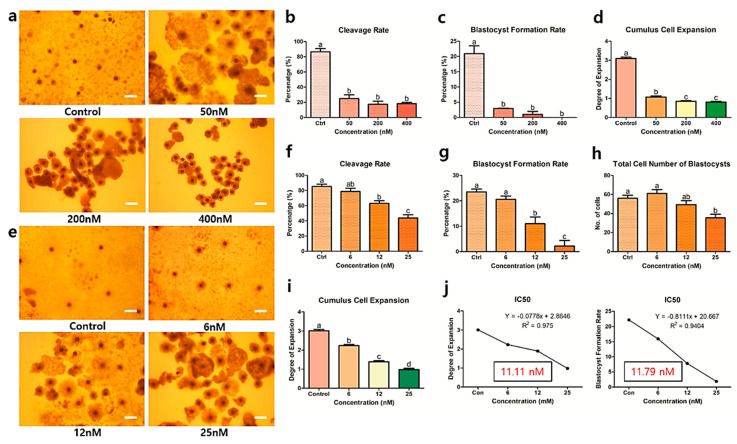
Selection of optimal brusatol concentration via the assessment of embryo development and cumulus expansion. Brusatol concentrations: 0, 50, 200, and 400 nM (first concentration range); 0, 6, 12, and 25 nM (second concentration range). (**a**) Microscopy-based images of cumulus cell expansion in porcine COCs treated with the first concentration range of brusatol are shown; (**b**–**d**) subsequently, embryo development and CC expansion rates were evaluated. (**e**) A subsequent analysis using the second concentration range of brusatol was performed. Acceptable results were observed for (**f**) cleavage rate, (**g**) blastocyst formation rate, (**h**) total cell number, and (**i**) CC expansion. (**j**) Lastly, the selected brusatol concentration (12 nM) was confirmed by calculating the IC_50_ (11.11 and 11.79 nM, respectively). Bars with letters indicate significant differences (*p* < 0.05). Original magnification: 20×; bars indicate 300 μm. Ctrl or Con: control. At least five replicates were performed for each experiment.

In order to determine the appropriate concentration range of brusatol for IVM, we first employed a range used in a previous study [8] (50, 200, and 400 nM); control COCs were treated with DMSO. Figure 1a shows morphological changes in CC expansion after 44 h of IVM and after PA. A significant and sharp decrease in cleavage rate (24.84%, 17.39%, and 18.24% vs. 86.67%, respectively, *p* < 0.05) and blastocyst formation rate (2.93%, 0.98%, and 0% vs. 20.95%, respectively, *p* < 0.05) was observed upon treatment with 50, 200, and 400 nM brusatol (as compared to the control group; Figure 1b,c). Moreover, a similar decrease was observed in CC expansion in the groups treated with 50, 200, and 400 nM brusatol compared to the control (Degree 1.07, 0.84, and 0.80 vs. Degree 3.094, respectively, *p* < 0.05) (Figure 1d).

Therefore, we used lower concentrations (0, 6, 12, and 25 nM; Figure 1e) of brusatol and found that treatment with brusatol at concentrations of 12 and 25 nM resulted in a significant decrease in the cleavage rate (63.08% and 43.87% vs. 85.40% and 78.65%, respectively, *p* < 0.05) and blastocyst formation rate (11.02% and 2.21% vs. 23.52% and 20.62%, respectively, *p* < 0.05) as compared with those observed in the control—6 nM brusatol (Figure 1f,g). Furthermore, 25 nM brusatol significantly reduced the total cell number of blastocysts when compared to that in the control and 6 and 12 nM brusatol-treated group (35.40 vs. 56.10, 61.11, and 49.29, respectively, *p* < 0.05). Figure 1h,i show that 6, 12, and 25 nM brusatol caused significant decreases in the CC expansion rate when compared to the control (Degree 2.23, 1.39, and 0.98 vs. Degree 3.01, respectively, *p* < 0.05). Additionally, based on the CC expansion and blastocyst formation rates, the chosen concentration (12 nM) was confirmed by calculating IC_50_ with the rate of CC expansion and blastocyst formation, which was determined to be approximately 11.11 and 11.79 nM brusatol (r^2^ value: 0.975 and 0.9404, respectively, *p* < 0.05) (Figure 1j). Because the IC_50_ values were close to the chosen concentration, 12 nM brusatol was selected as the optimal concentration for the subsequent experiments.

### 3.2. Effects of Melatonin on Oocyte Maturation and Subsequent Embryo Development

**Figure 2:**
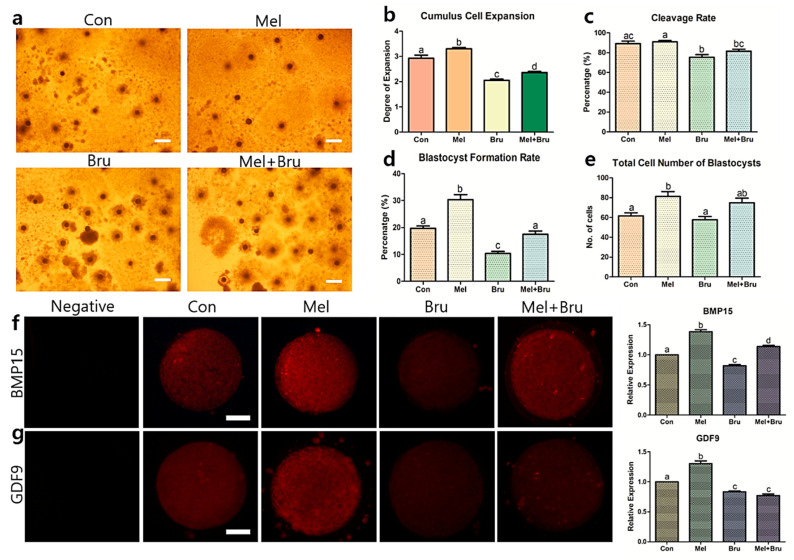
Evaluation of treatments with melatonin and brusatol on porcine COCs and their competences. With the treatment of 10^−9^ M melatonin and 12 nM brusatol, their effects on the CC expansion and subsequent embryonic development were evaluated. The four experimental groups (control, melatonin, brusatol, and co-treatment) showed distinct differences in (**a**,**b**) the CC expansion, (**c**) cleavage rate, (**d**) blastocyst formation rate, and (**e**) total cell number. Additionally, protein expressions related to competence of the porcine COCs from each experimental group were evaluated by immunocytochemistry. The protein expression levels in (**f**) BMP15 and (**g**) GDF9 showed significant differences among the groups. Bars with letters indicate significant differences (*p* < 0.05). 20× magnification, bars indicate 300 μm in Figure 2a and 400× magnification, bars indicate 30 μm in Figure 2f,g. At least three replicates were performed for each experiment.

Along with the chosen concentration of brusatol (12 nM), 10^−9^ M melatonin was used for oocyte maturation. Four groups, the control, melatonin, brusatol, and co-treatment with melatonin and brusatol (co-treatment) groups, were created for subsequent experiments (Figure 2a). First, the CC expansion rate in each experimental group was evaluated. The melatonin-treated group showed the highest CC expansion rate when compared to the control, brusatol, and co-treated groups, as shown in Figure 2b (Degree 3.30 vs. Degree 2.92, 2.05, and 2.36, respectively, *p* < 0.05). Notably, all experimental groups exhibited significant differences in the CC expansion rate. During embryo development, the control and melatonin-treated groups showed higher cleavage rates compared to the brusatol and co-treated groups (89.16% and 90.96% vs. 75.25% and 81.44%, respectively, *p*< 0.05) (Figure 2c). Moreover, the melatonin-treated group showed a higher blastocyst formation rate compared to the control, brusatol, and co-treated groups (30.36% vs. 19.71%, 10.35%, and 17.43%, respectively, *p* < 0.05) (Figure 2d), while the brusatol-treated group showed the lowest rate of blastocyst formation. Finally, the melatonin-treated group also exhibited the highest total cell number in blastocysts among the control, brusatol, and co-treated groups (81.20 vs. 61.60, 57.80, and 74.90, respectively, *p* < 0.05) (Figure 2e).

In addition, we evaluated oocyte maturation-related factors (BMP15 and GDF9) that are only expressed in oocytes (Figure 2f,g). BMP15 levels were the highest in the melatonin-treated group and lowest in the brusatol-treated group (*p* < 0.05). Moreover, the co-treated group exhibited restored BMP15 protein expression when compared to the brusatol-treated group. The same pattern was observed for the protein expression of GDF9, except for the co-treated group, which showed no significant difference as compared to the brusatol-treated group (*p* < 0.05). In summary, we partially verified that melatonin positively affected on oocyte maturation and embryo development; this was in agreement with the results of previous studies.

### 3.3. ATP Production, GSH/ROS Levels, and Gene Expression Patterns in COCs

**Figure 3:**
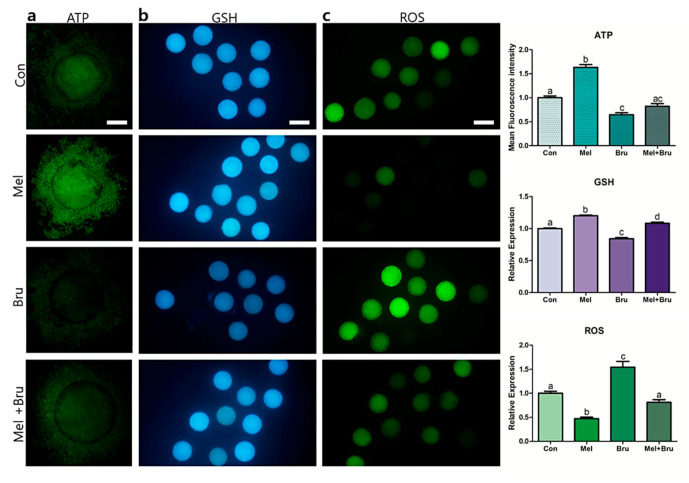
Determination of ATP production and Glutathione (GSH) and reactive oxygen species (ROS) levels in porcine oocytes. The four experimental groups (control, melatonin, brusatol, and co-treatment) showed significant differences in (**a**) ATP production (BODIPY staining), (**b**) GSH level (CellTracker Blue staining), and (**c**) ROS level (H2DCFDA fluorescence). Bars with letters indicate significant differences (*p* < 0.05). Figure 3a: 200× magnification; bars indicate 50 μm. Figure 3b,c: 100× magnification; bars indicate 100 μm. Con: control, Mel: melatonin, Bru: brusatol, and Mel + Bru: melatonin+brusatol. At least three replicates were performed for each experiment.

The ATP production, GSH/ROS levels, and gene expression patterns in porcine oocytes were assessed in subsequent analyses. When compared to that in the other groups, ATP expression was significantly increased in the melatonin group (*p* < 0.05) and was lowest in the brusatol-treated group (Figure 3a). As shown in Figure 3b, all experimental groups exhibited significant differences in GSH levels. In particular, as compared to that in other groups, the GSH level was significantly increased in the melatonin-treated group and it was the lowest in the brusatol-treated group; furthermore, the GSH level was recovered in the co-treated group compared to that in the brusatol-treated group (*p* < 0.05). Figure 3c depicts the ROS levels in oocytes, which were lowest in the melatonin-treated group and highest in the brusatol-treated group (*p* < 0.05). Lastly, the level of ROS in the co-treated group was significantly lower than that in the brusatol-treated group, which indicated that melatonin could overcome the effect of brusatol (*p* < 0.05).

Next, we evaluated the overall gene expression patterns in COCs. The total RNA was extracted from the COCs, purified, and trimmed (Tables S1–3). Figure S1a shows raw data of read counts for each experimental group. To filter the data, a logarithm was applied, followed by trimming by TMM normalization. In addition, Figure S1d shows the correlation of each experimental group after normalization; notably, clear differences were observed between the control vs. brusatol treatment. Moreover, through multidimensional scaling, the closest relationship was observed between the control and melatonin-treated groups, and the brusatol- and co-treated groups showed larger differences than expected (Figure S1b). Prior to DEG analysis, quality control was performed, and we found that 16,720 genes were mapped across all of the samples (Figure S1c); these data were used for subsequent DEG analysis.

### 3.4. Analysis from DEG and Gene Ontology (GO) Term in Porcine COCs

**Figure 4:**
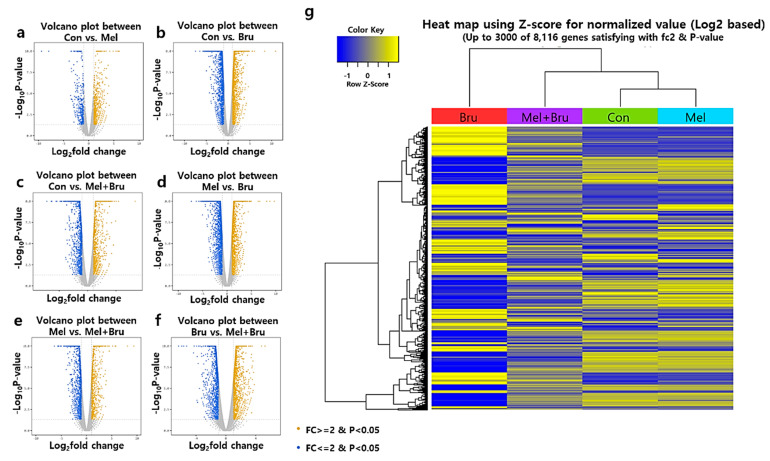
General gene expressions in porcine COCs from the four experimental groups (control, melatonin, brusatol, and co-treatment). (**a**–**f**) Total of six cases of gene expression comparisons were compared: Con vs. Mel, Con vs. Bru, Con vs. Mel + Bru, Mel vs. Bru, Mel vs. Mel + Bru, and Bru vs. Mel + Bru. Yellow dots indicate expression changes of up-regulated genes that have more than 2-fold higher with *p* < 0.05, and blue dots indicate expression changes of down-regulated genes that have more than two-fold lower with *p* < 0.05. (**g**) A heat map was generated using the normalized value from each experimental group, it showed up to 3000 of 8116 genes satisfying with two-fold changes and *p* < 0.05. Con; control, Mel; melatonin, Bru; brusatol, and Mel + Bru; melatonin+brusatol. At least 1100 porcine COCs per experimental group were collected from eight biological replications.

**Figure 5:**
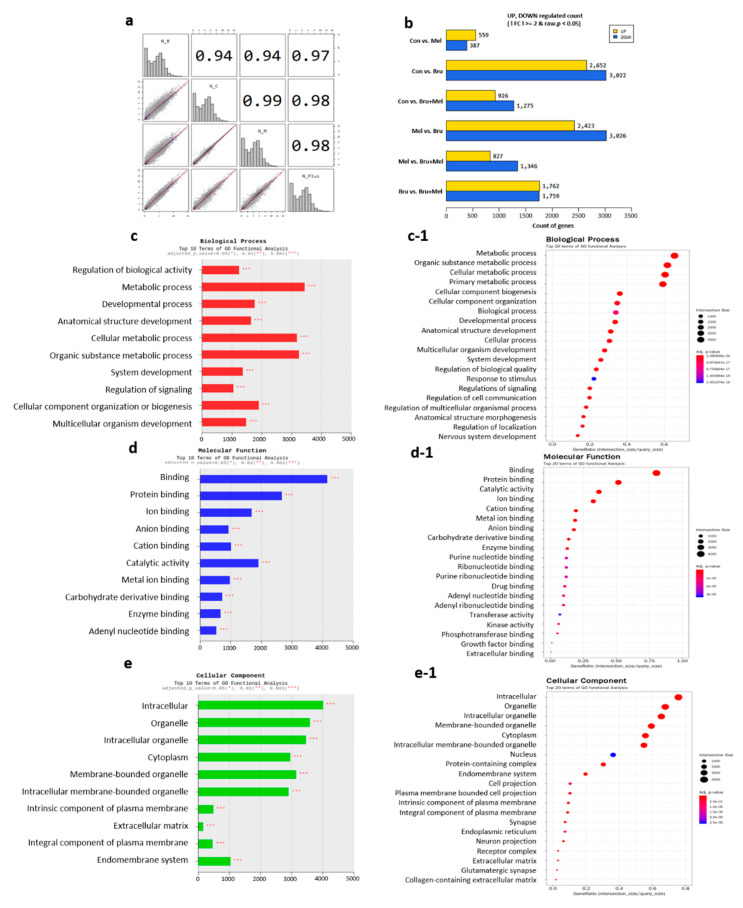
Evaluation of sample reproducibility and results of the top 10 terms of GO functional analysis. (**a**) Pearson’s coefficient was tested to validate the reproducibility among the samples and (**b**) numeric interpretations of differences in DEGs were demonstrated, compared to six cases within the experimental groups: Con vs. Mel, Con vs. Bru, Con vs. Me l+ Bru, Mel vs. Bru, Mel vs. Mel + Bru, and Bru vs. Mel + Bru. (**c**–**e**) Top 10 GO terms were analyzed in ‘biological process’, ‘molecular function’, and ‘cellular component’, and (**c-1**–**e-1**) top 20 terms of GO functional analysis were demonstrated by the size of intersection. Con; control, Mel; melatonin, Bru; brusatol, and Mel + Bru; melatonin + brusatol.

We analyzed DEG and GO term with the trimmed data in order to confirm differences of genetic expression levels among the experimental groups. Figure 4a–f show comparisons of gene expression levels of individual groups, respectively. Figure 5b demonstrates numeric interpretation, and the biggest difference in DEGs was shown in control versus brusatol-treated group, consistent with Figure 4b. Furthermore, other Figures also showed significant differences. Figure 4g summarizes the overall DEG patterns among the groups. Interestingly, the co-treated group had the most diverse patterns compared to other experimental groups, and brusatol-treated group had an opposing DEG pattern when compared to that of the control and melatonin-treated groups. With the normalized data, we applied Pearson’s coefficient in order to verify reproducibility among the samples (Figure 5a); looking at the correlation coefficients, we found that control and melatonin-treated group versus brusatol-treated group had the least similarity and control and melatonin-treated groups were the most similar. Indeed, these values are verified in pigs, as values below 0.70 indicate differences in species. Figure 5c–e showed significant changes in biological process, molecular function, and cellular component within all of the experimental groups. In biological process, ‘metabolic process’ was the most significant enrichment term among the 10 terms and ‘developmental process’ also had a *p*-value less than 0.001 (Figure 5c). As the result, the processes that are related to metabolism, biogenesis, or development had the most significant changes in COCs in accordance with Figure 5c-1. Interestingly, in ‘cellular component’ (Figure 5d,d-1), the most frequent and significant changes were observed in ‘organelle’ and ‘intracellular’ section and genes related to peroxisome and oocyte were detected (*p* < 0.05). Finally, in ‘molecular function’, functions related to ‘binding’ were the most significant enrichment terms; in addition, we found genes related to oocyte maturation, embryo development, apoptosis, peroxisome, and antioxidant response. Subsequent analysis was performed with the genes of interest from these data.

### 3.5. Gene and Protein Expression Related to Melatonin-Nrf2 Signaling and Peroxisome Mechanism

**Figure 6:**
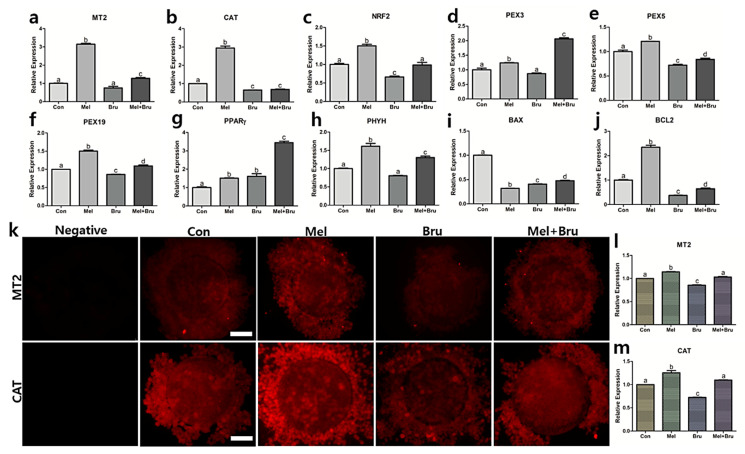
Assessments of genes of interest by qRT-PCR and protein expression analysis by immunocytochemistry in porcine COCs. (**a**) Gene expressions related to (**a**,**b**) melatonin and antioxidant (*MT2* and *CATALASE*), (**c**) Nrf2 signaling (*NRF2*), (**d**–**h**) peroxisomal activities (*PEX3, PEX5, PEX19, PPARγ*, and *PHYH*), and (**i**,**j**) apoptosis (*BAX* and *BCL2*) were tested. (**k**–**m**) The protein expression of MT2 and CATALASE in porcine COCs were evaluated by immunocytochemistry. Bars with letters indicate significant differences (*p* < 0.05). Original magnification: 400×, bars indicate 30 μm Con; control, Mel; melatonin, Bru; brusatol, and Mel + Bru; melatonin + brusatol. At least three technical and biological replicates were performed.

**Figure 7:**
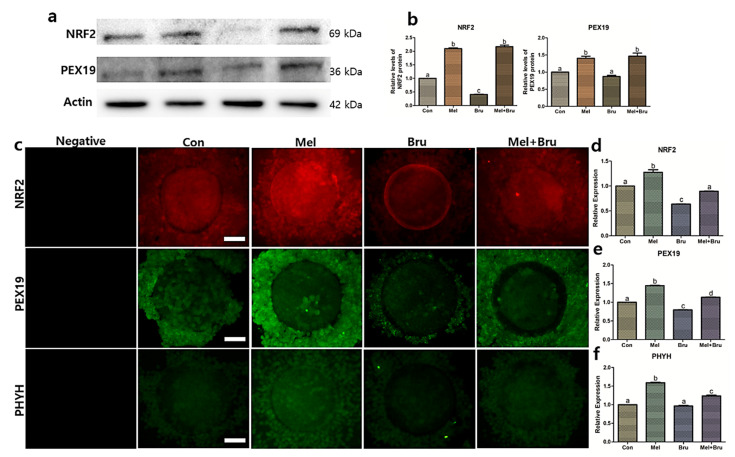
Protein analysis of protein of interest in porcine COCs by immunocytochemistry and western blotting. (**a**,**b**) Western blot was performed to analyze and quantify NRF2 and PEX19 proteins in the porcine COCs. Beta-actin was used as the reference protein. (**c**–**f**) In order to re-validate the proteins expressed in the COCs, immunocytochemistry was performed. Bars with letters indicate significant differences (*p* < 0.05). Original magnification: 400×, bars indicate 30 μm Con; control, Mel; melatonin, Bru; brusatol, and Mel + Bru; melatonin + brusatol. At least three technical and biological replicates were performed.

Factors that were related to melatonin-Nrf2 signaling and peroxisome were evaluated. Melatonin receptor 2 (MT2) had the highest gene expression level in melatonin-treated group (Figure 6a) as well as protein expression level in Figure 6l. The protein expression level of MT2 in brusatol-treated group was significantly lower than the control, but not in gene expression. In case of catalase, the gene and protein expressions were the highest in melatonin-treated group (Figure 6b,m). The results of immunocytochemistry (ICC) of MT2 and catalase are shown in Figure 6k–m. With regards to gene expression in PEX3, the co-treated group was significantly higher than other experimental groups, and the melatonin-treated group was also significantly higher than control and brusatol-treated groups (Figure 6d). In PEX5, the gene expression levels of each experimental group showed significant differences. Melatonin group had the highest expression level among the groups and the co-treated group was significantly higher than brusatol-treated group (Figure 6e). The gene expression of PPAR*γ* in the co-treated groups was the highest among the groups and the melatonin- and brusatol-treated groups were significantly higher than the control (Figure 6g). When including the melatonin-treated group, the gene expression in BAX for all of the groups was significantly lower than the control group, while the expression of BCL2 in the melatonin-treated group was more than two-fold higher than the other groups (Figure 6i,j). The melatonin-treated group was the highest gene expression in NRF2 compared to other experimental groups; the brusatol-treated group was significantly lower than the control. In addition, the co-treated group was significantly higher than brusatol-treated group (Figure 6c). A similar pattern was observed in the protein expression levels of NRF2, verified through western blot analysis and ICC (Figure 7a,c). Specifically, the result of western blotting in NRF2, the expression level of the co-treated group was the same as that of the melatonin-treated group (Figure 7b). In PEX19, all of the experimental groups had significant differences except for brusatol-treated group and the highest expression level in the melatonin-treated group (Figure 7b). Additionally, the expression level of the co-treated group was significantly higher than that of the brusatol-treated group. The protein expression of PEX19, as verified by ICC, had the same pattern as the gene expression pattern that is shown in Figure 6f; however, western blot analysis showed that the co-treated group showed no difference compared to the melatonin-treated group. Lastly, gene and protein expression levels of PHYH had the same expression patterns (Figure 6h and Figure 7f), with the melatonin-treated group having the highest expression level compared to the other groups, and the co-treated group was significantly higher than the control and brusatol-treated groups.

## 4. Discussion

To our knowledge, we have demonstrated, for the first time, the possibility of a crosstalk between melatonin-Nrf2 signaling and peroxisomal activities in porcine COCs and partially validated these findings using RNA sequencing. Our results suggest that peroxisomes may crosstalk with melatonin via the Nrf2/ARE signaling pathway, as most of the related mRNA and protein expression levels were significantly up- or down-regulated, and the expansion of CCs and embryo development altered in response to treatment with melatonin and brusatol in the porcine COCs. In addition, catalase activity and ATP production were also differently regulated by the treatments, which implied that the antioxidant mechanism and energy metabolism were influenced by the crosstalk between NRF2 signaling, and peroxisomes in accordance with our hypothesis.

**Figure 8:**
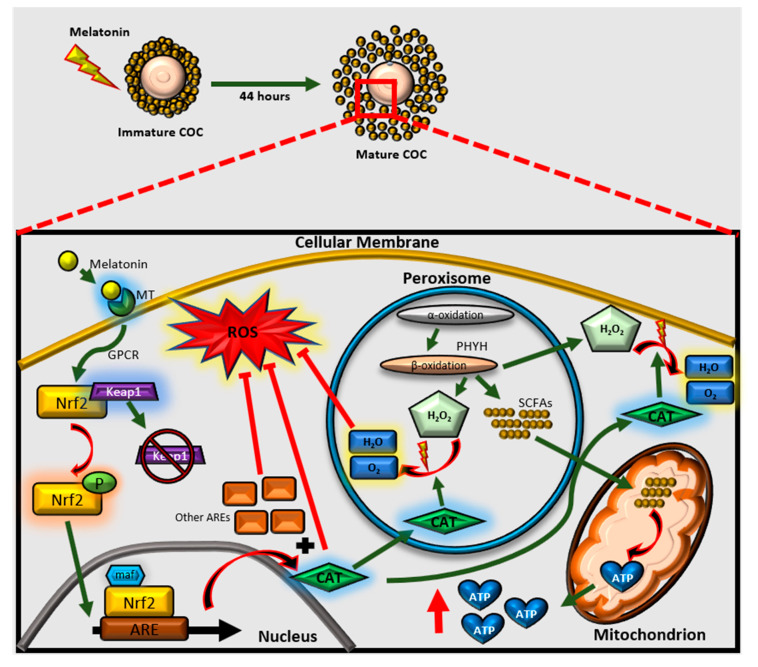
A schematic illustration of the cross-talk between Nrf2 signaling and peroxisomes activated by melatonin in porcine COCs. When melatonin binds to melatonin receptor 2, a series of cascades is activated via the GPCRs. Nrf2 is subsequently activated by the detachment of Keap1, which is later targeted for degradation. Activated Nrf2 is translocated to the nucleus, and transcription is initiated in conjunction with maf. Catalase is produced that prevents ROS accumulation, hydrolyzes H_2_O_2_, and also work in peroxisomes. Through α- and β- oxidation, Short-chained fatty acids (SCFAs) and H_2_O_2_ are produced in the peroxisome. The SCFAs are transferred to mitochondria for energy production. In addition, catalases are also stored in the peroxisome to hydrolyze H_2_O_2_ generated during peroxisomal activities. MT: melatonin receptor.

Figure 8 shows a schematic of this study. Our study shows that melatonin can improve porcine oocyte quality and subsequent embryonic development through its various biological activities [10,40,41]. Melatonin is thought to mediate its effect by binding to receptors on the membrane, i.e., MTs [42], which constitute a superfamily of guanine nucleotide binding protein (G-protein)-coupled receptors [43,44]. Specifically, MT2 was found to be fundamentally involved in oocyte maturation, embryo development, and the activation of antioxidant-related signaling in porcine oocytes and embryos [8,40]; accordingly, our results showed that the regulation of MT2 resulted in differences in mRNA and protein levels among the treatment groups. As demonstrated in many studies, melatonin is known to regulate the Nrf2/ARE signaling pathway [8,11,12]; therefore, we employed brusatol, a Nrf2 inhibitor, in order to verify the mechanism.

No CC expansion was observed with high concentrations of brusatol (50, 200, and 400 nM) and lower concentrations (12 and 25 nM) also showed negative effects on CC expansion (Figure 1). This corroborated our hypothesis that brusatol can specifically inhibit Nrf2 signaling and could influence the CC expansion in COCs. Kwak et al. suggested that the down-regulation of Nrf2 in oocytes may decrease CC expansion [45], which supported our results in that visible reductions in CC expansion, including the reduction in subsequent embryonic development, were clearly observed.

Upon evaluating embryonic development in the control, melatonin treatment, brusatol treatment, and co-treatment groups, we found that the brusatol-treated group had the lowest developmental potential during the embryonic stage. This result is supported by a study by Ma and Lin et al., who demonstrated a significant decrease in the maturation of mouse oocytes and embryonic development following brusatol treatment [17,46]. In addition, our results are similar to those that were obtained in previous studies [9,10,40] in that melatonin not only increased subsequent embryo development, but also increased the maturation rates of porcine COCs. Moreover, the expression levels of GDF9 and BMP15, which are related to oocyte competence [47], were also significantly influenced in each experimental group. Various studies also suggested that melatonin is associated with ROS mechanisms in oocytes [48,49,50], and our results corroborated these findings. Additionally, we observed the negative effects of brusatol on GSH and ROS levels. This may be due to the fact that brusatol, a Nrf2 inhibitor, could affect not only embryonic development, but also oxidative stress. In other words, embryonic development can also be decreased due to a negative redox imbalance due to brusatol, which is supported by the report by Guerin et al. that the generation of ROS impairs embryonic development in various ways [51]. In summary, we partially demonstrated the actions of melatonin and brusatol via Nrf2 signaling in pigs; therefore, we subsequently conducted intracellular studies in the COCs.

Interestingly, the expression pattern of DEGs showed drastic differences in the brusatol- and co-treated groups when compared to that in the control and melatonin-treated groups. This may imply that various causes are responsible for the impairment of COC morphology. First, as brusatol was confirmed to decrease CC expansion and hamper embryo development, the Nrf2 signaling pathway may strongly affect the morphological changes and developmental potential in porcine COCs; this is similar to the results obtained in previous studies [17,46]. Second, ATP content was significantly decreased in the brusatol-treated group and it was increased in response to melatonin treatment in the COCs. Because ATP is produced in the mitochondria and peroxisomes mainly though lipid β-oxidation [28], we hypothesized that melatonin and brusatol can also affect peroxisomes and mitochondria via the Nrf2 signaling pathway. The results of GO term analysis revealed meaningful functions, such as ‘in the peroxisome’, ‘peroxisomal fission’, ‘the PPAR signaling pathway’, ‘mitochondrion’, ‘mitochondrial membrane’, ‘mitochondrial translational elongation’, etc., which were all included in the top 10 GO terms in cellular components (Figure 5e,e-1). Moreover, we found significant differences in ‘ATP synthesis coupled electron transport’, ‘ATP metabolic process’, ‘ATPase activity’, and ‘antioxidant activity’, all of which were also included in top 10 GO terms in molecular functions. It has been suggested that SCFAs that are produced during α- or β- oxidation in peroxisomes are transferred to the mitochondria for subsequent ATP production [52]. Furthermore, considering the fact that ATP can be produced by the peroxisomes themselves [53] and that ATP from FA oxidation enhances oocyte competence [54,55], we narrowed our study in order to examine the effects of peroxisomes and their relationship with the Nrf2 signaling pathway.

In order to track the enzymatic activities in peroxisomes, we used PHYH and PEX19. PHYH is involved in α-oxidation, which is thought to occur only in the peroxisomes [52]. Therefore, we surmised that PHYH would serve as a useful peroxisome marker. More specifically, PEX19 is one of the major factors associated with peroxisomal membrane biogenesis, and plays numerous roles in the functional assembly of the peroxisome [56]. Our data showed that peroxisomal activities were significantly increased in the melatonin-treated group and decreased in the co-treated group, which was verified by the levels of PHYH and PEX19. This may suggest that melatonin could increase peroxisomal activities, which could be reduced upon co-treatment with brusatol. In other words, we suggest the possibility that Nrf2 signaling regulates peroxisomal activities. This is corroborated by a study by You et al., which showed that knockdown of *PEX19* caused a reduction in peroxisome number, resulting in increased apoptosis and simultaneous reduction in the activation of Nrf2 signaling and facilitation of ROS accumulation [57]. Our results regarding the NRF2 expression levels support the findings of You et al., in that similar expression patterns were observed for NRF2 when compared to those of PHYH and PEX19.

Another connection between Nrf2 signaling and peroxisomes can be explained by catalase activation. Catalase exists within and outside of the peroxisome, and plays a major role in maintaining the oxidative balance of the organelle by breaking down hydrogen peroxide produced in the peroxisome [27,58]. Catalase is also known as a peroxisomal marker protein that is associated with PEX19 activity [59]. Miller et al. demonstrated that a significant reduction in catalase levels occurred in *Nrf2* knockout mice [60] and a similar phenomenon was observed in pigs [61]. Together with previous reports, our data also showed that the protein expression pattern of catalase was similar to that of Nrf2 and PEX19 in that the melatonin-treated group, which exhibited significantly increased expression of catalase; additionally, catalase expression in the co-treated group was significantly lower than that in the melatonin-treated group, which demonstrated an alternative connection between Nrf2 signaling and peroxisomes.

## 5. Conclusions

In conclusion, the upregulation of Nrf2 signaling and peroxisomes by melatonin treatment followed by their downregulation by brusatol imply a strong relationship between them; this is depicted in Figure 8. To our knowledge, this is the first study to examine the collaborative roles and functions in porcine oocytes activated by melatonin. Because antioxidant mechanisms and energy production are involved in oocyte maturation and subsequent embryonic development in mammals, further studies are essential in order to uncover the intricate crosstalk between Nrf2 signaling and the peroxisomes.

## Figures and Tables

**Table 1:** Sequence-specific primers used for quantification of differential expressed transcripts.

Genes	Primer Sequences (5′–3′)	Product Size (bp)	Accession No.
*GAPDH*	F: GTCGGTTGTGGATCTGACCT R: TTGACGAAGTGGTCGTTGAG	207	NM_001206359
*MT2*	F: AGCTGCCTTAACGCCATCAT R: ATTGTCGCCCAGTCAGTGAG	219	XM 021063941.1
*catalase*	F: AGGGAGAGGCGGTTTATTGC R: GGACTCGTTGGTGAAGCTCA	117	NM_214301.2
*NRF2*	F: GCCCAGTCTTCATTGCTCCT R: AGCTCCTCCCAAACTTGCTC	115	XM_013984303
*PEX3*	F: AATGCATCTTCCTGGGGACG R: ATACTGTCGTCGTGCTTGGG	125	NM_001244185.1
*PEX5*	F: CAGGCGGAGAATGAGCAAGA R: GGACTCGTTGGTGAAGCTCA	117	XM_013988424.2
*PEX19*	F: CCAGCACTTCACCCATCAGT R: TAGACGACACTCCTGCCTCA	144	XM_001928869.5
*PPARγ*	F: CTCAATCTATCGGGCCCACC R: TTTATCCCCACAGACACGGC	192	XM_005669783.3
*PHYH*	F: CCCTTCAGGCCCAGCAATAA R: GCCTTTGTGAGTTCCTGGGA	102	NM_001113447.1
*BAX*	F: CATGAAGACAGGGGCCCTTT R: CATCCTCTGCAGCTCCATGT	181	XM_003127290
*BCL2*	F: AATGTCTCAGAGCAACCGGG R: GGGGCCTCAGTTCTGTTCTC	193	NM_214285

F, Forward primer; R, Reverse Primer.

## Supplementary Materials

The following are available online at https://www.mdpi.com/2076-3921/9/11/1080/s1, Figure S1 Raw data and trimmed read counts in porcine COCs from RNA sequencing, Table S1 Raw data of sequencing from mature COCs, Table S2 Trimmed data of sequencing from mature COCs, Table S3 Result of mapped data from cDNA fragments.
