# Supplementary material for: Melatonin-Nrf2 Signaling Activates Peroxisomal Activities in Porcine Cumulus Cell-Oocyte Complexes
Source: Antioxidants (Basel). 2020 Nov 3;9(11):1080. doi: 10.3390/antiox9111080 (PMC7692444; doi:10.3390/antiox9111080)
Supplement: Supplementary file 1 [file antioxidants-09-01080-s001.pdf]

Supplementary material

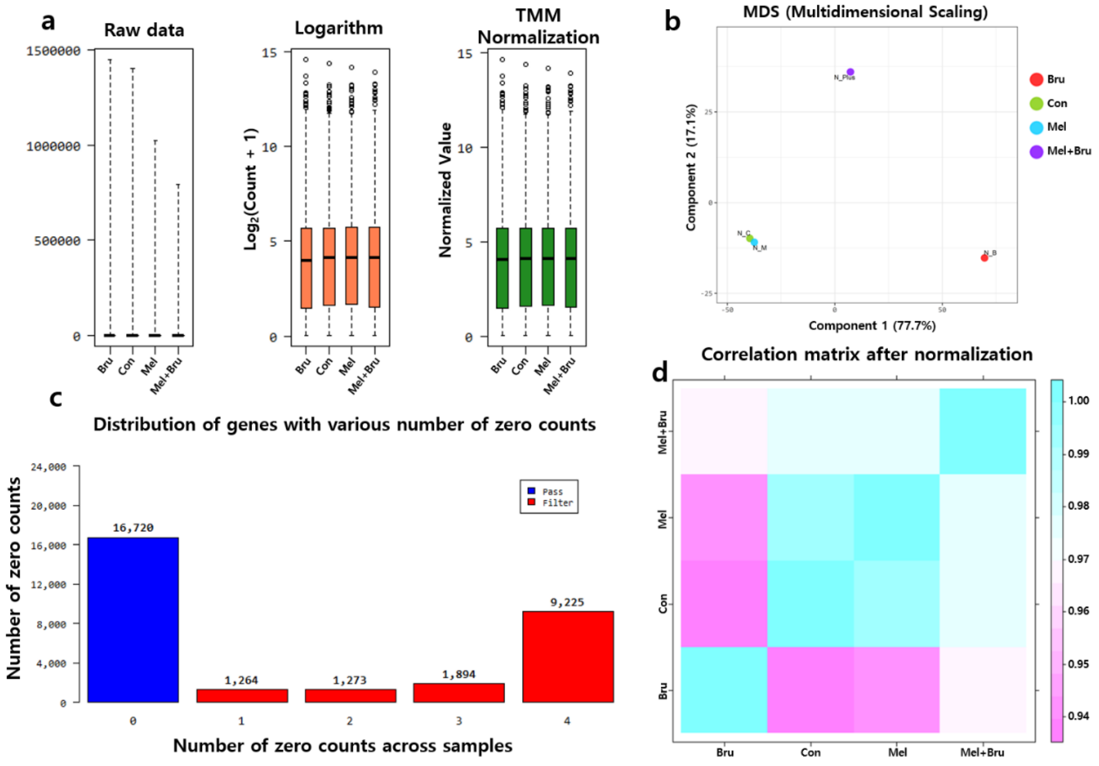

Figure 1. Raw data and trimmed read counts in porcine COCs from RNA sequencing. (a) the raw data from the COCs were processed by a series of data trimming up to data normalization, applying logarithm and TMM normalization. (b) The normalized data was first used for multidimensional scaling and (c) the quality control was performed for analyzing DEGs, then as the result, total 16,720 genes were mapped. (d) Lastly, a correlation among the genes were demonstrated as a matrix after data normalization. Con; control, Mel; melatonin, Bru; brusatol, and Mel+Bru; melatonin+brusatol.

Table 1. Raw data of sequencing from mature COCs.

Group	Total Read Bases*	Total Reads	GC (%)	Q20 (%)	Q30* (%)
Control	8,351,726,158	82,690,358	52.03	97.95	94.27
Melatonin	6,910,083,872	68,416,672	51.83	98.21	94.93
Brusatol	7,250,491,242	71,787,042	48.24	98.29	94.80
Melatonin+Brusatol	6,150,647,904	60,897,504	51.65	98.23	94.81

* Total read bases - Calculated by ‘Total reads × Read length’.* Q30 (%) : Ratio of bases that have better quality than Phred quality score 30.

Table 2. Trimmed data of sequencing from mature COCs.

Group	Total Read Bases*	Total Reads	GC (%)	Q20 (%)	Q30* (%)
Control	8,136,781,932	81,169,626	52.02	98.58	95.12
Melatonin	6,746,869,394	67,271,268	51.84	98.80	95.72
Brusatol	7,121,678,665	70,894,536	48.24	98.71	95.39
Melatonin+Brusatol	6,027,897,479	60,032,956	51.66	98.72	95.49

* Total read bases - Calculated by 'Total reads × Read length'.* Q30 (%) : Ratio of bases that have better quality than Phred quality score 30.

Table 3. Result of mapped data from cDNA fragments.

Group	Number of processed reads*	Number of mapped reads* (%)	Number of unmapped reads (%)
Control	81,169,626	79,230,444 (97.61)	1,939,537 (2.39)
Melatonin	67,271,268	65,674,842 (97.63)	1,596,426 (2.37)
Brusatol	70,894,536	69,635,999 (98.22)	1,258,537 (1.78)
Melatonin+Brusatol	60,032,956	58,858,650 (98.04)	1,174,306 (1.96)

* Processed reads : Number of cleaned reads after data trimming.* Mapped reads : Number of mapped reads according to the genomic DNA reference (GCF_000003025.6_Sscrofa 11.1).
